# How Many Glucan Chains Form Plant Cellulose Microfibrils? A Mini Review

**DOI:** 10.1021/acs.biomac.4c00995

**Published:** 2024-08-29

**Authors:** Daniel J. Cosgrove, Paul Dupree, Enrique D. Gomez, Candace H. Haigler, James D. Kubicki, Jochen Zimmer

**Affiliations:** † Pennsylvania State University, University Park, Pennsylvania 16802, United States; ‡ Department of Biochemistry, University of Cambridge, Cambridge CB2 1QW, United Kingdom; § Crop Sciences and Department of Botany, North Carolina State University, Raleigh, North Carolina 27695, United States; ∥ Department of Geological Sciences, UTEP University of Texas El Paso, El Paso, Texas 79968, United States; ⊥ Molecular Physiology and Biological Physics, University of Virginia, Charlottesville, Virginia 22903-1738, United States

## Abstract

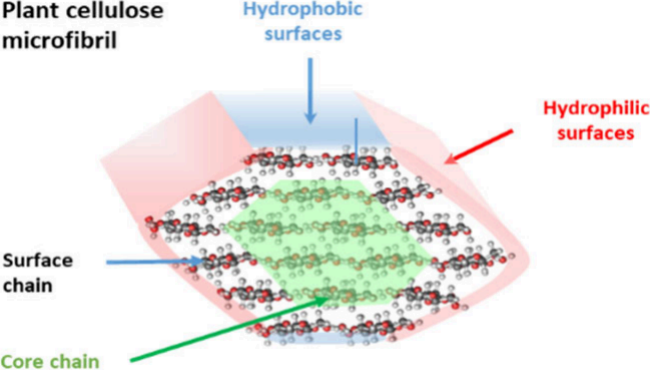

Assessing the number of glucan chains in cellulose microfibrils (CMFs) is crucial for understanding their structure–property relationships and interactions within plant cell walls. This Review examines the conclusions and limitations of the major experimental techniques that have provided insights into this question. Small-angle X-ray and neutron scattering data predominantly support an 18-chain model, although analysis is complicated by factors such as fibril coalescence and matrix polysaccharide associations. Solid-state nuclear magnetic resonance (NMR) spectroscopy allows the estimation of the CMF width from the ratio of interior to surface glucose residues. However, there is uncertainty in the assignment of NMR spectral peaks to surface or interior chains. Freeze-fracture transmission electron microscopy images show cellulose synthase complexes to be “rosettes” of six lobes each consistent with a trimer of cellulose synthase enzymes, consistent with the synthesis of 18 parallel glucan chains in the CMF. Nevertheless, the number of chains in CMFs remains to be conclusively demonstrated.

## 1 Introduction: Significance of CMF Chain Number and Shape

Cellulose has vast economic value in the form of building materials (e.g., in wood products), textile fibers (e.g., cotton which is nearly pure cellulose), forage for livestock, feedstocks for bioenergy, diverse polymers and chemicals, and in the burgeoning field of nanocellulosics. As the most abundant form of organic matter in organisms on Earth, cellulose plays a central role in the global-scale storage and cycling of photosynthetically captured CO_2_, which gains importance for attempts to counteract the environmental effects of fossil fuel combustion. In land plants, cellulose is synthesized by a multiprotein cellulose synthase complex (CSC), resulting in a long fibril of ∼3 nm width that is commonly called the cellulose microfibril (CMF). The CMF is an integral structural component of nearly every cell wall in the body of plants and is a key determinant of many physical, chemical, biological, and structural properties of cell walls.^1−3^ The terminology surrounding CMFs and their potential supramolecular assemblies sometimes varies between research groups and across disciplines; therefore, we define our terms in Table 1. Further explanation and references follow within the text.

**Table 1:** Definitions Applicable to Land Plants with Rosette CSCs

cellulose protofibril	The product of three oligomerized cellulose synthases, such as formed by recombinantly expressed CesAs or within one lobe of the rosette CSC. The three β-1,4-linked d-glucosyl polymers are aligned in parallel.
cellulose microfibril (CMF)	A linear crystalline cellulose-I fibril synthesized by one rosette CSC.
macrofibril	The product of the stable association of multiple CMFs and other cell wall polymers, e.g., matrix polysaccharides and lignin in woody cell walls.
fiber	A single elongated plant cell characterized by a thick and strong, secondary cell wall assembled on a scaffold of cellulose microfibrils and, optionally, macrofibrils. Examples include cotton fibers, wood fibers, ramie, flax, and hemp.

**Figure 1:**
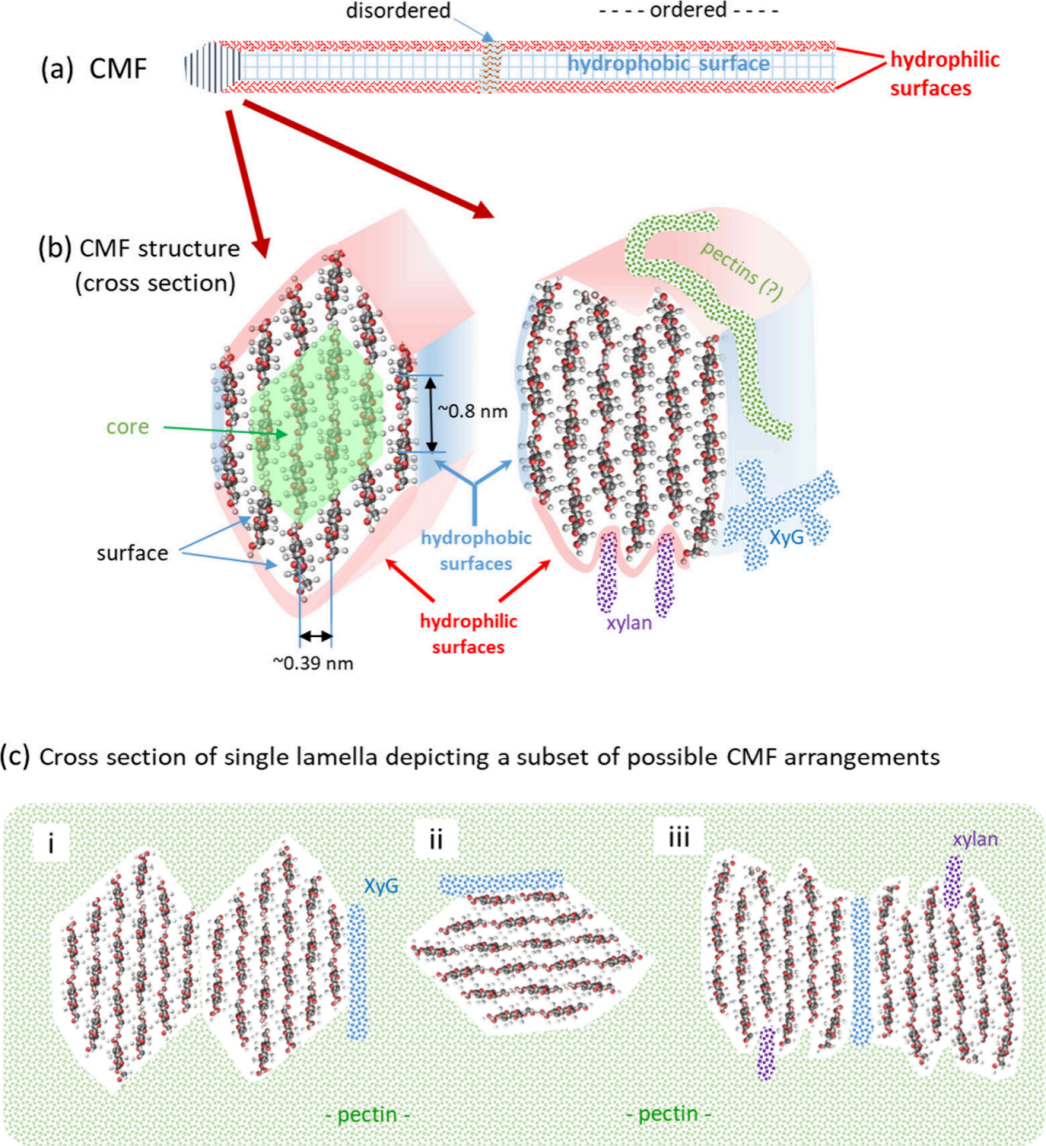
Features of the cellulose microfibril (CMF) structure and organization. (a) CMFs are relatively stiff rod-like structures with high order (crystallinity) and perhaps some disordered (noncrystalline) regions, but the location and proportion of any disordered cellulose within CMFs is uncertain. Surface chains are more mobile than core chains. CMFs have multiple faces with distinct hydrophilic and hydrophobic properties. (b) Possible models of CMFs made of 18 glucan chains are illustrated in cross sections with hexagonal and approximately rectangular habits (shapes). Other habits may also be possible. The habit determines the proportion of the different CMF surfaces that are relatively hydrophilic vs hydrophobic. The distance between the “sheets” of cellulose chains is ∼0.4 nm and between chains within a sheet the distance is ∼0.8 nm. Xyloglucan (XyG; blue) is hypothesized to bind to the hydrophobic surfaces, whereas xylan is hypothesized to bind to the hydrophilic surfaces. The internal core chains in the left fibril are shaded green. (c) Hypothetical arrangements of CMFs and the matrix in an abbreviated cross section of a single cell wall lamella. In primary cell walls, CMF regions may be found as “singletons” (ii) or in bundles of two or more CMFs laterally bound to each other, without (i) or with (iii) intermediary xyloglucan. In this illustration, CMF–CMF binding occurs via the hydrophobic surfaces, but other studies suggest hydrophilic surfaces also mediate bundling. In secondary cell walls, xyloglucan is absent, and bundling into macrofibrils with several CMFs may involve xylan and lignin interactions. Images a, b, and d were adapted with permission from ref (2). Copyright 2018 Elsevier.

Selected aspects of the CMF structure are illustrated in Figure 1. The number of chains in the CMF cross section, their packing, and the CMF cross-sectional shape likely affect its physical and chemical properties, as well as its interfacial interactions with other CMFs, matrix polymers, and water.

Knowing these details of CMF structure is important for attempts to construct physically accurate models of CMF properties such as strength, elasticity, plasticity, thermal and wetting behavior, chemical reactivity, and enzyme susceptibility, as well as for models of plant cell walls and materials derived from them.

**Figure 2:**
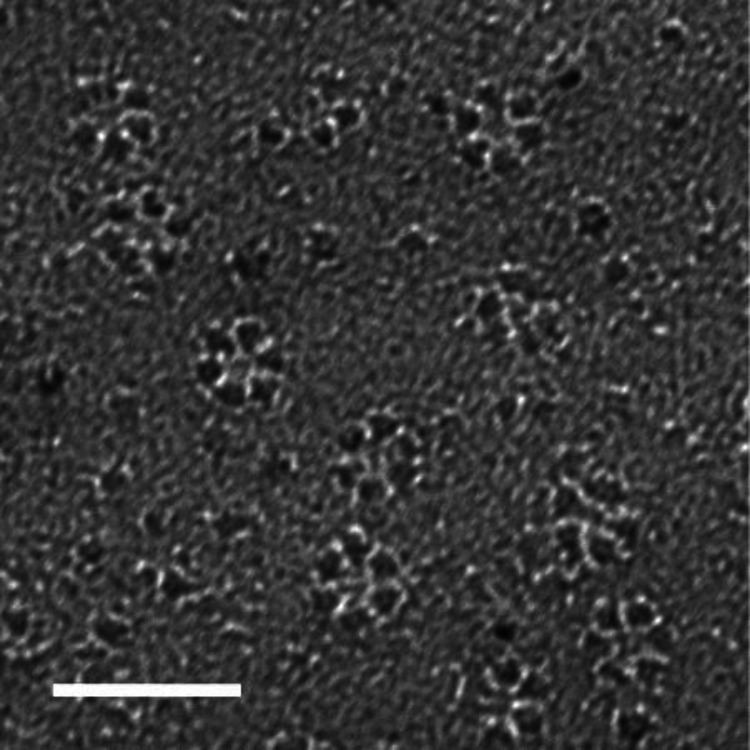
Hexameric rosette CSCs in the plasma membrane of a cell synthesizing a secondary cell wall. A sample containing differentiating tracheary elements of *Zinnia elegans* was frozen, fractured, shadowed, and replicated prior to TEM imaging. The resolved transmembrane regions of oligomerized CesAs are organized into six lobes in each CSC and visualized top-down. The cytosolic (catalytic) parts of the CSCs were below the plasma membrane and were not visible. The scale bar is 100 nm. (Image provided by R.L. Blanton, NC State University.)

Central to all of this is an accurate count of the number of parallel chains in the CMF, which remains controversial. The number has conventionally been approximated by fitting of molecular models of cellulose crystal structures^4,5^ to CMF dimensions as well as by estimating the number of CesAs in CSCs. In recent literature, it is common to find CMFs modeled with 36, 24, or 18 chains in cross section,^6−13^ based on results from a variety of approaches. The chain numbers are commonly multiples of six because of the hexameric appearance of CSCs in freeze-fracture images^14^ (Figure 2), corresponding to, respectively, a theoretical possibility of 6, 4, or 3 synthases in each of the hexameric repeat units. However, other chain numbers have also been modeled, e.g., see ref (12). The newest advance in CMF chain counting takes into consideration the organization of CesAs into trimeric complexes, suggesting that CSCs are hexamers of CesA trimers.^15−17^

Most recent studies have tended to favor 18 or 24 chains for the CMF. Here we assess the information from physical and biological approaches and their limitations in estimating the number of cellulose chains in the CMF. The information comes from microscopy, X-ray and neutron scattering, solid-state NMR (ssNMR), quantum modeling, and CSC architecture.

## 2 Microfibril Diameter from High-Resolution Microscopy

In primary and secondary cell walls of land plants, estimates of CMF width based on TEM images commonly range from 2.5 to 4.0 nm.^18^ An electron tomography study of delignified pine wood rendered a CMF width of ∼3.2 nm, but the authors did not address the chain number.^19^ Cryo-electron tomography of onion epidermal walls visualized irregular “fiber bundles” ranging from 5 to 6 nm in diameter.^18^ With a maximal resolution of 3.8 nm, individual CMFs with potentially 18 or 24 chains could not be resolved in this study, and the “fiber bundles” were assumed to be composed of at least two CMFs. After negative staining for TEM, microfibrils observed in vitro in experiments with recombinant plant cellulose synthases have ranged in width from 2–3 nm^20^ to ∼5 nm.^21−23^

Atomic force microscopy (AFM) has also been used to examine the CMF width in isolated CMFs and in whole walls. Early AFM images of walls detected CMFs *in situ*, but relatively large AFM tips tended to overestimate CMF widths, e.g., refs (24−26). More recent studies using finer AFM tips and more advanced instrumentation have reported individual CMF diameters of ∼3.5 nm with evidence of extensive bundling.^8,27^ Microfibril dimensions in maize primary cell walls were reported to be ∼3.7 nm wide and 2.25 nm high, which the authors viewed as consistent with an 18-chain CMF.^8^ For comparison, in the crystal structures of cellulose Iβ, the individual glucan chains are stacked with a spacing of 0.4 nm and separated laterally by 0.8 nm^5^ (Figure 1).

Tight binding of hemicelluloses to native CMF surfaces may exaggerate CMF diameters, as would tight lateral adherence (bundling) of CMFs. Hence, AFM provides an upper estimate of the CMF diameter, with the smallest fibril diameters as the likeliest candidates for bare, single CMF dimensions.

## 3 Scattering from Plant Cell Walls Is Most Consistent with an 18-Chain Model for CMFs

Wide-angle and small-angle scatterings are sensitive to CMF dimensions. Reports of the CMF diameter from wide-angle X-ray scattering (WAXS) or small-angle X-ray or neutron scattering (SAXS or SANS) are often near 2.5–4 nm.^28^ In principle, scattering techniques can be quite sensitive to small variations in the diameter of a cylindrical object, such that accurately revealing microfibril dimensions to an accuracy commensurate with the width of a glucan chain (ca. 0.5 nm) should be possible. Recent simulations of the expected scattering from 14-, 18-, 23-, 28-, 34-, and 40-chain CMFs explicitly show how various observables from wide- and small-angle X-ray scattering can differentiate between these different models.^12^ In practice, various factors can limit the accuracy needed to address the number of chains within cellulose microfibrils.

One approach for estimating the diameter of a CMF is by analyzing the crystal coherence length obtained from WAXS. The crystal coherence length, obtained through a Scherrer analysis,^29,30^ is often taken to be the crystal size or width. Formally, the Scherrer equation reveals the length scale over which the coherence in lattice planes is lost, which can be limited by the size of the crystal or cumulative (paracrystalline) disorder.^31,32^ If cumulative disorder dominates, then the coherence length becomes a lower bound on the crystal size. Nevertheless, accurate estimates of the coherence length require a value for the shape factor (often termed K), which can vary from 0.62 to 2.08.^33^ The K shape factor for estimating the cellulose crystal coherence length is currently unavailable, although a value of 0.9 is often assumed and leads to estimates that are close to the CMF diameters obtained from SAXS (∼3 nm)^34^ and would be consistent with an 18-chain microfibril model.^12^ It is thus currently difficult to use the coherence length obtained from WAXS peak widths to accurately estimate CMF dimensions, although such estimates have been reported.^35^

SAXS or SANS can also reveal the CMF dimensions. A useful way to describe small-angle scattering data is that it is composed of the product of form and structure factors, which describe the resulting interference that arises from intradomain and interdomain correlations, respectively. For dense systems, isolating scattering from either the structure or form factor is rather challenging. Indeed, proteins in solution must be studied at low concentrations, or correlations between proteins in solution dominate the scattering pattern.^36,37^ For plant cell walls, the high density of CMFs suggests that structure factors should contribute substantially to the scattering data.^38^ Careful analyses of small-angle X-ray scattering data from Norway spruce wood reveal contributions from the spatial arrangement of microfibrils, or the structure factor, if the form factor is properly deconvoluted from the data.^39^ SAXS from celery^40^ and SANS from Sitka spruce wood^41^ also reveal scattering corresponding to the spacing between CMFs (interdomain correlations), which changes with the degree of hydration. Obtaining an accurate upper estimate of the CMF diameter is likely possible by analyzing the interdomain spacing obtained from scattering data and assuming that CMFs exist in a dry bundle. It is highly likely, however, that some matrix polysaccharides, or perhaps amorphous glucan chains, lie between microfibrils, even when bundled. This would result in an overestimation of the CMF diameter when extracting this length scale from correlation peaks in small-angle scattering data.

**Figure 3:**
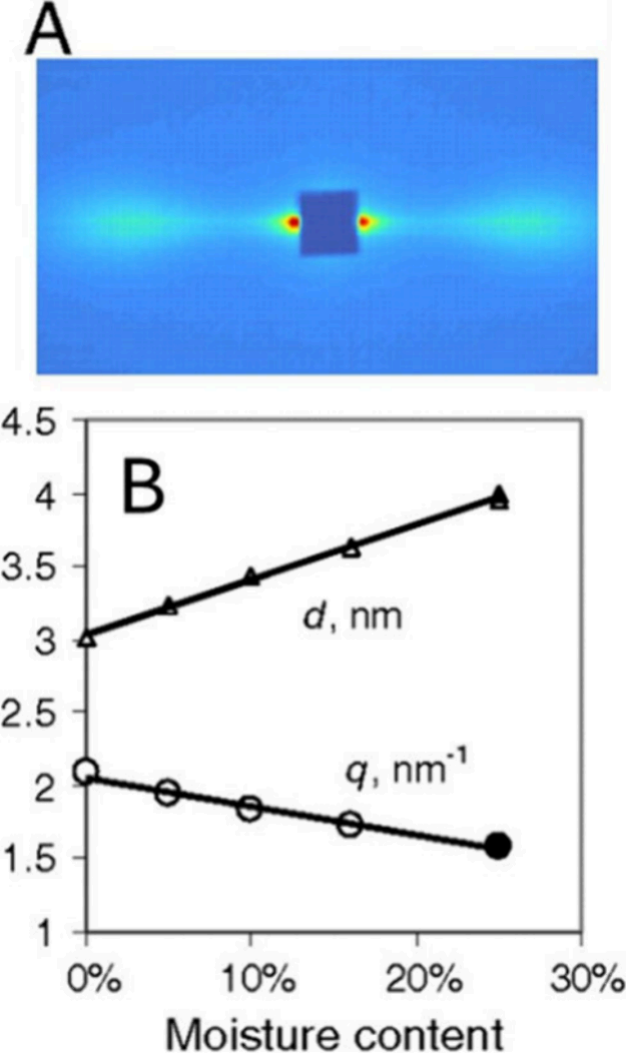
(A) SANS data from spruce wood showing two equatorial peaks corresponding to the spacing between CMFs. (B) Spacing from SANS as a function of the moisture content. The increase in spacing (d) with water absorption suggests that there are hydrophilic chains in between CMFs, placing an upper bound of the CMF diameter to less than 3 nm. Reproduced with permission from ref (41). Copyright 2011 PNAS.

Given the substantial number of reports of CMFs with diameters between 2.5 and 3 nm^28^ from SAXS and SANS, it is challenging to imagine how CMFs with 24 chains (and potentially surface-bound matrix polysaccharides) can fit within this length scale. Indeed, simulations of SAXS data from an 18-chain model reveal scattering-derived expected diameters of about 2.6 nm for bare CMFs and 3.1 nm for CMFs with surface-bound hemicellulose.^12^ In addition, careful SANS measurements of spruce wood clearly show a 3 nm center-to-center spacing between dry CMFs, which places an upper limit on the CMF diameter (Figure 3). Furthermore, including the likely possibility of a hemicellulosic polysaccharide chain (such as glucomannan and xylan) between microfibrils would explain the ability of CMF spacing to swell to 4 nm with hydration. These observables are challenging to reconcile with a 24-chain CMF.^41^

## 4 What Can ssNMR Resolve?

Solid-state NMR (ssNMR) is widely used to estimate the fibril dimensions. The most frequent estimates of 18–24 chains are broadly consistent with the other methods discussed here.^42,43^ The ssNMR method relies on the observation that the ^13^C chemical shifts of C atoms in cellulose glucosyl residues are sensitive to their environment within the fibril. Notably, glucosyl residues have carbon 4 (C4) shifts in either the 89 or 84 ppm regions. Glucose residues with C4 shift around 89 ppm are commonly considered crystalline and internal (core) to the fibril, but they are also known as D1 glucose residues to avoid a firm assignment to a fibril location. On the other hand, residues with C4 shift around 84 ppm are considered to exist on the fibril surface or within amorphous cellulose, and these are also known as D2 glucose residues. For the purposes of CMF diameter estimation, the ratio is calculated of the signal from D1:D2 glucose residues (D1/D2), and this allows an estimate of the fibril interior/surface ratio.^42^ Nevertheless, there are many difficulties in the accurate measurement of this ratio, and there are also uncertainties about the interpretation of these signals, because they may not arise solely from fibril interior and surface glucose residues. If we accept the assumptions and the data are optimally acquired, then models of the CMF can be assessed since, for example, thinner fibrils will have a greater surface to interior ratio. Nevertheless, the ratio does not alone give a clear prediction of total number of chains without also considering the possible range of CMF habits (arrangements of the chains in the fibril).^44^

There are difficulties in the accurate measurement of the ratio of the glucose residue environments in CMFs. The cellulosic glucose C4 chemical shift is widely used because the C4 spectral peaks are relatively well resolved from other components in the complex mixture of the plant wall and, therefore, can be quantitated from 1D ssNMR spectra. Nevertheless, there are other non-CMF components that contribute to the C4 spectral peak estimates, particularly in the D2 region in spectra of the whole cell wall material. For example, some of the signals from lignin, pectin, and hemicellulosic components (arabinan, glucomannan, and 2-fold xylan) can contribute to the spectrum. This problem can be reduced by the subtraction of some of the signals arising from these components since they may be more mobile than the CMFs.^42,45^ The D2 region may also have contributions from noncrystalline fibril glucans, including what is sometimes called “amorphous” or disordered cellulose. Indeed, the D1/D2 ratio is also often used to calculate a “crystallinity index” of cellulose materials, where ordered surface and other glucans contribute to the D2 signal (e.g.,^46^). The noncrystalline cellulose signals are found in pulped cellulosic materials.^47,48^ Although these polymers have not been clearly described in intact cell walls, the biochemical and wood literature suggests the presence of some “amorphous” or “para-crystalline” glucans in the wall, such as acid hydrolyzable glucan.^49^ These may be glucans synthesized by different biosynthetic machinery to the canonical rosette CSCs (such as CesAs in different arrangements) or components arising from changes to the CMFs during cell wall assembly or during extraction and preparation of the sample. Such nonfibrillar glucan chains in the sample will contribute to the D2 region, leading to an overestimation of surface and an underestimation of CMF size.

To expedite data acquisition and improve sensitivity, cross-polarization (CP) NMR is usually used to acquire cellulose spectra. In these CP experiments, magnetization is transferred to the carbon nuclei from nearby protons. The signal strength depends on the motion of the residues, so CP spectra reveal the relatively immobile components in the sample, including CMFs, whereas highly mobile components are not visible. However, it is not clear that all glucose residues in the CMF proportionally contribute to the CP spectra, as surface residues have motion somewhat greater than that in the CMF core. The data acquisition parameters will affect the relative strength of signals reported for D1 and D2 glucosyl residues.^50^ To address this problem, multiple acquisitions of ssNMR data have been carried out to determine the extent of the phenomenon and the D1:D2 ratio calculations adjusted accordingly.^6^ Nevertheless, it is difficult to know how effectively this approach can accommodate the motion of several different environments within cellulose.

**Figure 4:**
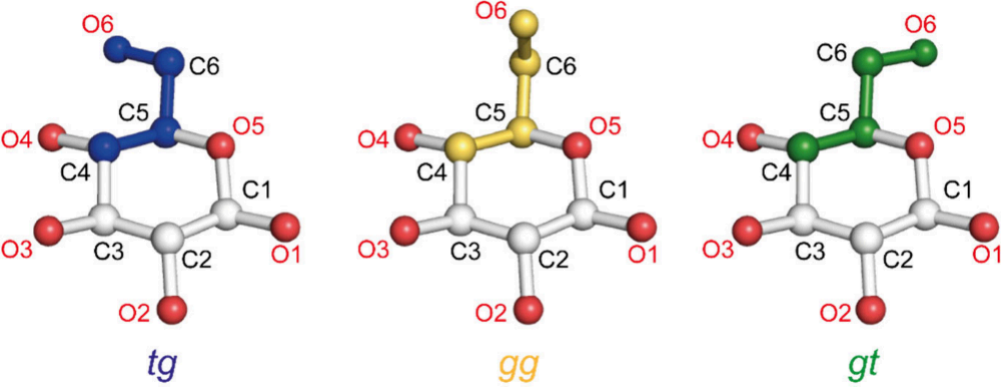
Illustration of the three conformations of the C6 hydroxymethyl group of glucose residues in cellulose. The *tg* conformation has a C4 chemical shift around 89 ppm (D1), whereas the *gg* and *gt* conformation glucose residues have a C4 chemical shift around 84 ppm (D2).

There are further assumptions in the fibril diameter calculations from the D1:D2 ratio. There is a good experimental basis of the D1 and D2 assignment, largely to interior and surface residues, respectively, based on solvent accessibility experiments.^47,51,52^ However, it remains unclear if D1 environments are entirely interior of the fibril. To understand why there is uncertainty, we need to consider the basis of the different NMR shifts in D1 vs D2. The ssNMR shift does not directly reflect interior versus surface residues. These differences in the C4 shifts arise largely because of the different C6 hydroxymethyl conformations in the glucosyl residues. The C6 hydroxymethyl O is closer to the C4 carbon in the *tg* hydroxymethyl conformation (Figure 4), leading to a signal in the D1 region.^11,53^

The *tg* conformation is favored when the glucosyl residue C6 hydroxymethyl is stably hydrogen-bonded, such as in the crystal interior, and hence, these core residues have largely D1 chemical shifts. Most surface residues are in the *gt* and *gg* conformations, where the C6 hydroxymethyl groups are bonded to water and are more mobile. The ssNMR measured ratio of D1 and D2 environments is essentially measuring the *tg* to *gt*/*gg* C6 hydroxymethyl conformation ratio. In assigning D1 and D2 to interior and surface, the assumption is that all internal residues are *tg* and all surface residues are *gt*/*gg*. However, atomistic simulations suggest some surface residues facing inward to the fibril core will have a *tg* conformation^44,54^ and several experimentalists noted that D1 may include a component of surface residues.^45,53,55^ Thus, by assigning all D1 to the interior of the fibril, the proportion of the surface may be underestimated and fibril size can be overestimated. Indeed, the estimate of 24 chains in ref (6) may be an overestimate in part because it relied on the fibril core, as measured by scattering to be equivalent to the D1 residues.

NMR is an averaging technique of all fibrils in a sample, so any variability in the fibril dimensions will be averaged. If there is partial coalescence, as proposed, for example, in cotton and flax fibrils, an average dimension of the small and larger bundled fibrils will be calculated. Some broad D2 peaks arise from aggregated fibrils in pulped material, with a heterogeneous interaction surface environment and, hence, range of C4 shifts in D2.^47,56^ The influence of this phenomenon on the estimation of CMF D2 abundance has been partly overcome by spectral deconvolution to remove broad peaks of aggregated cellulose. Another issue is that coalescence of fibrils may also convert surface environments to “interior” D1 signals in order to explain the relatively high proportion of D1 in wood. For example, it has been suggested that fibrils may form bundles where fibril D2 surface environments will be converted to apparently interior D1 signals.^57^ Surface residues may also be measured within D1, for example, when bonding to hemicellulose such as xylan, where they may adopt a *tg* conformation.^55,58^ Without knowing the extent of fibril coverage by hemicellulose and the extent of fibril coalescence in the sample, an estimation of fibril dimensions by ssNMR is, therefore, likely to be inaccurate. In recent years, 2D ssNMR has revealed many different glucosyl residue environments in CMFs in both D1 and D2.^52,55,59^ This technique has the potential to clarify fibril dimension assessment, but will require all of these environments to be assigned to specific regions of the CMF, especially to determine which are surface versus internal glucose residues.

## 5 Insights from CSC Architecture

**Figure 5:**
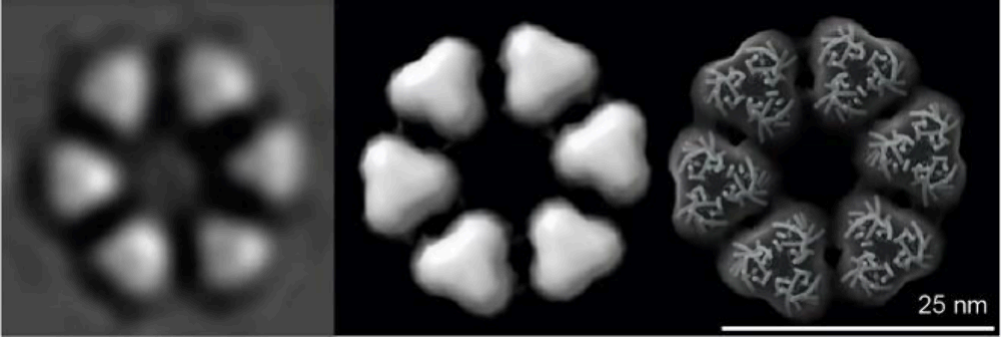
Comparison of an image average of a CSC structure with a theoretical model assembled from CesA trimers. Left: The image average is reprinted with permission under a Creative Commons license CC BY 4.0 from ref (17). Middle: Envelope of the transmembrane region of a theoretical CSC assembled from CesA trimers (PDB: 6WLB). Right: The semitransparent envelope is shown together with a cartoon representation of the transmembrane segments of the assembled CSC.

Assembling cellulose polymers into a CMF likely requires the close association of multiple cellulose-producing CesAs into a supramolecular complex.^60^ These complexes, commonly referred to as CSCs or rosettes, have been visualized by freeze fracture TEM (FF-TEM) in several species^14,61−64^ (Figure 2). The technique provides architectural information about the transmembrane regions of integral membrane proteins, such as CesAs. Accordingly, in land plants and certain green algae, CSCs appear as an approximately hexagonal arrangement of six repeat units, and they have been labeled in situ with antibodies to CesAs.^65^ The CSCs have an overall mean diameter of approximately 21 to 23 nm,^17^ and the six repeats (called lobes) often roughly resemble an equilateral triangle of about 8 nm in length (Figure 5).

Earlier observations of CSCs and microfibrils by FF-TEM supported a model of 36 CesAs forming a CSC and, accordingly, 36 cellulose chains constituting an “elementary” CMF.^62,64^ To the contrary, the possibility that a CSC contained no more than 24 CesAs was suggested based on estimates of how many transmembrane helices could fit within each lobe of the rosette.^66^ This number was reduced to 18 based on *in silico* modeling approaches with a partial CesA structure that included a complete set of seven transmembrane helices as well as most of the enzyme’s cytosolic domains.^17^ It was concluded that the size of a CSC lobe as visualized by FF-TEM is most consistent with it containing three CesAs, resulting in 18 enzymes per CSC, organized into a “hexamer of trimers” (Figure 5).^17^

Cryogenic electron microscopy structures of CesAs from hybrid aspen and cotton (CesA8 and CesA7, respectively) support this interpretation.^15,16^ The structures revealed homotrimeric 3-fold symmetric assemblies of the synthases, with no evidence of higher oligomeric states of the recombinantly expressed and purified enzymes. Further, the trimer’s transmembrane region reflects in size and shape the dimensions of a CSC lobe.^15^

Combined, structural biology and modeling approaches provided strong support for a “hexamer of trimer” organization of CesAs within a rosette CSC.^15,17^ Accordingly, its CMF product would consist of a maximum of 18 glucan chains, if all CesAs were active.^44^ Within a CesA trimer, concurrently synthesized and secreted glucan chains are released about 3 nm apart from each other, which may facilitate their coalescence into a protofibril of three chains. Neighboring protofibrils of a CSC, in turn, would emerge at a distance of about 10 nm from each other and their alignment would give rise to an 18-chain CMF.^15^ FF-TEM images of membranes of land plants sometimes show twins, triplets, or loose clusters of CSCs,^67,68^ but the potential role of CSC aggregation in generating a subpopulation of larger CMFs is not clear (see further discussion in Section 7).

It is currently unknown whether CSCs can sometimes associate with additional CesA subunits to generate complexes of more than 18 CesAs and thus form fibrils with more than 18 cellulose chains. Conceivable arrangements may include the association of one or two catalytically active CesA trimers with a CSC and the alignment of the produced protofibrils with an 18-chain elementary fibril to generate a 21 or 24 chain CMF, respectively. However, this would have to be a linearized and uniform accretion process, since the surfaces of CMFs observed in AFM appear smooth within the resolution limits of the technique (see, for example, ref (69)). Any contribution of non-CSC CesAs to cellulose deposition in the cell wall remains to be determined.

## 6 Insights from Quantum Mechanical Models

Quantum mechanical modeling can be used to test various interpretations of CMF size and habit by comparing the calculated properties of CMF models to observations. Kubicki and co-workers have been testing and refining models of Iα and Iβ cellulose using density functional theory (DFT) to calculate structures, energies, vibrational frequencies (IR, Raman and SFG), and ^13^C NMR chemical shifts that correlate well with observations.^70−73^ Generally, the quantum mechanical approach is more accurate than force field-based classical molecular simulations and provides a direct route to predicting spectroscopic parameters, such as ^13^C NMR chemical shifts, that are central to the debate regarding CMF size and habit.

**Figure 6:**
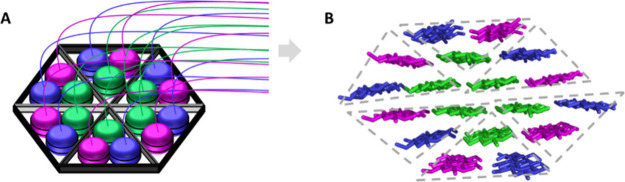
Correlation between CSC structure and predicted CMF structure. (A) Cartoon of a rosette CSC with 3 CesAs within each of 6 lobes. Each CesA is assumed to be active, synthesizing one glucan chain. (B) Cross-section of an 18-chain CMF model with a 2–3–4–4–3–2 habit (note the number of glucan chains in each horizontal layer). The overall arrangement of glucan chains (within six boxes with dashed borders) mimics the arrangement of CesAs within the CSC. Reproduced with permission from ref (44). Copyright 2020 Springer Nature.

Models were constructed to mimic CMFs rather than the periodic crystalline models of earlier studies.^73^ Following the work in refs (17 and 74), CMF models were based on 18-cellulose chains that would be produced by the hexamer of trimers in the CSC. Three arrangements of the CMFs were considered reasonable based on arrays of 6 layers of 3 chains, a 2–3–4–4–3–2 grouping, and a 3–4–4–4–3 CMF with 5 layers. Comparing modeled energies, δ^13^C values, and WAXS diffractograms, the 3–4–4–4–3 CMF was determined to be slightly favorable over the 2–3–4–4–3–2 structure and the 6 × 3 model as improbable. However, these CMFs were in vacuum, so they did not include H-bonding effects from water or any matrix polysaccharides. In ref (44) this was rectified, as monolayers of H_2_O molecules were added to surround the CMFs. In addition, variations on the interior and exterior hydroxymethyl torsions were investigated in order to better resolve ^13^C NMR spectra assigned to internal and external C6 exocyclic groups (e.g., ref (52)). This study concluded that the 2–3–4–4–3–2 arrangement was most probable based on agreement with observed ^13^C NMR spectra and relative energies. Notably, a model with all surface cellulose chain C6 exocyclic groups in the *gt* conformation was significantly higher in energy than the model with C6 pointed toward the interior in *tg* conformation. Only the C6 groups pointed away from the CMF should be in *gt* conformation whereas C6 groups pointed toward the interior of the CMF (even though the cellulose chain was on the surface) were more stable in the *tg* conformation as interior cellulose chains. Thus, basing the number of cellulose chains in a CMF on the assumption that all surface chain C6 groups are in *gt* conformation is inconsistent not only with NMR data as described above but also with the DFT results (e.g., ref (6)). Furthermore, the 2–3–4–4–3–2 habit was shown to be consistent with formation from a CSC with a hexamer of CesA trimers (Figure 6).

## 7 Biological Variability

Given the strong evidence that each rosette CSC synthesizes one 18-chain CMF, do these individual microfibrils undergo higher order interactions to form larger fibrils in the native cell walls? The answer is “yes” for macrofibrils such as those found in woody cell walls. These are about 10 to 60 nm in cross-section and composed of CMFs, hemicellulose (e.g., xylan and galactoglucomannan), and lignin. Macrofibrils have been analyzed by cryo-scanning electron microscopy, atomic force microscopy, electron tomography, NMR spectroscopy, and modeling *in silico*.^75−77^ Within these heterogeneous macrofibrils, the 18-chain CMFs are thought to retain their individual identity despite their close proximity. Similarly, the CMFs in primary walls remain distinct while they weave in and out of neighboring microfibril bundles.^45,69,78−80^

Can 18-chain CMFs coalesce and cocrystallize to form stable fibrils with greater cross-sectional dimensions and crystal width? The inherent properties of native cellulose allow it to undergo higher order self-assembly, as illustrated by classical examples across Kingdoms. Some bacteria arrange structurally and mechanistically homologous, but nontrimeric cellulose synthases into linear arrays, leading to the aligned glucan chains merging into smaller and then larger fibrils.^81−83^ Further, native cellulose in some marine algae can also assemble into extraordinarily large fibrils. For example, *Valonia macrophysa* synthesizes cellulose fibrils composed of 1200 to 1400 individual glucan chains that cocrystallize into a single, large (20 × 20 nm^2^) crystalline lattice.^84^ This correlates with the activity of a large rectangular cellulose synthesis complex with a quite different size and shape as compared to the rosette CSC (reviewed in ref (85)). Future research is needed to determine the suborganization of cellulose synthases within the linear or rectangular CSCs of diverse algae outside the Charophyceae and how this might contribute to the substructure of larger CMFs.^85^ In some Charophycean algae (e.g., *Micrasterias denticulata*), large, hexagonal arrays of rosette CSCs collectively synthesize banded cellulose fibrils.^63^ In *M. denticulata*, the variable width of each macrofibril correlates with the number of aligned CSCs contributing to its synthesis. Longitudinal substructure within these algal macrofibrils is consistent with each CSC synthesizing one 18-chain CMF that coalesced with its neighbors, but did not cocrystallize. In summary, these examples from bacteria and algae show that cellulose synthases can be assembled into various supramolecular arrangements to tailor the CMF properties.

Although many terrestrial plant species and cell types contain rosette CSCs,^14^ no large, ordered CSC arrays or highly organized two-dimensional arrays of CMFs have so far been observed. Do one or more nascent CMFs coalesce prior to crystallization in land plants so that large pure-cellulose fibrils exist in native cell walls? As reviewed previously,^86^ factors that could promote coalescence of two or more 18-chain CMFs during synthesis include: proximity of rosette CSCs in the plasma membrane (twins, triplets, and loose clusters occur, but singlets are most common); higher density of CSCs during secondary cell wall synthesis; preferential movement of CSCs in one direction; a poorly organized, hydrated shell of glucan chains on the microfibril surface; and a temporal gap between the secretion of cellulose protofibrils and their coalescence into fibrils. Alternatively, if the nascent CMF interacts with cellulose-interactive matrix polymers during synthesis, the individual CMFs would remain distinct although possibly bundled together. By analogy to bacterial cellulose synthesis, it is likely that cell wall matrix polymers^87^ can dynamically modulate the interactions of adjacent CMFs. When cellulose-binding polymers (including plant cell wall matrix polymers) are added to bacterial culture medium, cellulose self-assembly is hindered and composite materials with variable physical properties are formed.^88,89^ Complex molecular, electrostatic, biophysical, and energetic factors that are minimally understood are anticipated to impact the interactions of CMFs with themselves and/or other polymers in the wall.^87,90^

Cotton fibers are often characterized as having a larger cellulose crystal width than other cells or tissues in terrestrial plants (see ref (91) and references therein). These highly elongated and thickened seed epidermal cells are composed of about 95% cellulose. The outer primary walls contain cellulose and matrix polymers similar to other elongating plant cells, but no matrix component has been identified within the cellulose-rich secondary walls that compose most of the mature fiber.^92,93^ Cotton fibers of three *Gossypium* species have been analyzed in a study integrating X-ray diffraction, SAXS, SANS, ^13^C NMR, and Fourier transform infrared spectroscopy (FTIR).^94^ The data supported a model in which two or three 18-chain fibrils coalesced and cocrystallized to form a fibril with a 3.6–4.7 nm cross-section, approximately 2 nm crystalline core, and a “paracrystalline” shell that can be permeated by water.^94^ The formation of a large fibril from several 18-chain CMFs was attributed to the very low matrix content of cotton fiber secondary cell walls as compared to other plant cells.^94^ An analysis by WAXS and SAXS supported the idea that the CMFs in cotton fiber primary or secondary walls contain 36 or 72 glucan chains, respectively, whereas the CMFs in cotton stems contain 18 glucan chains.^95^ However, biophysical analysis of cotton fibers typically follows air drying or chemical dehydration of the fiber sample, and sometimes other chemical extractions are used as well.^91,94,96^ Drying or pretreatments could induce changes from the native state that were not recoverable, even if fibers are rehydrated for some analyses. In terms of understanding the native CMF structure and biological controls of cellulose and cell wall assembly, this deficit also applies to research on wood. Substantial dehydration occurs during wood lignification *in vivo*, and wood is often extracted and/or delignified prior to analysis of secondary cell wall nanostructure.

To resolve whether multiple 18-chain CMFs can cocrystallize *in vivo*, we need analytical tools that do not require drying or pretreatment of native cell walls. We need to understand when and where in the process of cell wall assembly 18-chain fibrils interact with their neighbors and matrix polymers and how these interactions are variably regulated between species, tissues, cell types, and developmental stages.^97^ Beyond CesAs, there are proteins with poorly understood roles that help to regulate cellulose synthesis and/or assembly.^97,98^ Delineating all components contributing to the cellulose biosynthesis *in vivo* will keep the community busy for the foreseeable future.

## 8 Conclusion and Perspectives

It seems most probable that many of the frequently observed rosette CSCs of land plants synthesize a single CMF with maximally 18 glucan chains. We cannot exclude the possibility that additional glucan chains synthesized by adjacent CesA trimers or alternative unknown ways coalesce onto the CMFs to create larger fibrils. At present, we are unable to determine the precise chain number in CMFs due to uncertainties in the biophysical measurements and the presence of confounding components in complex plant cell wall samples. Currently available scattering data appear most consistent with an 18-chain CMF. Computational modeling is a helpful tool to evaluate hypotheses about CMF structure. The use of 2-dimensional ssNMR to identify more precisely the proportions of glucosyl residues on the surface and the core of the CMF seems a promising route to estimate average dimensions of CMFs in samples. Isolation of CMFs without aggregation, hemicellulose coating, and confounding other cell wall glucan components for analysis by ssNMR will also yield clearer answers on the number of chains in the CMF. Looking to the future, it will be important to reconstitute CMF formation in vitro from purified components. Cryo-electron microscopy is a promising technique that might allow visualization of the chains in the isolated CMFs and resolve questions of chain number and arrangement (habits). It is important to know the habit of the CMFs to explain CMF bundling and the assembly of cellulose with other components of the cell wall. Then, we will be closer to understanding how CMFs contribute to the remarkable properties of cell walls, timber, and pulp.

## References

[ref1] Nishiyama Y. Structure and properties of the cellulose microfibril. J. Wood Sci. 2009, 55, 241–9. 10.1007/s10086-009-1029-1.

[ref2] Cosgrove D. J. Nanoscale structure, mechanics and growth of epidermal cell walls. Curr. Opin Plant Biol. 2018, 46, 77–86. 10.1016/j.pbi.2018.07.016. 30142487

[ref3] Jarvis M. C. Structure of native cellulose microfibrils, the starting point for nanocellulose manufacture. Philos. Trans A Math Phys. Eng. Sci. 2018, 376, 20170045 10.1098/rsta.2017.0045. 29277742

[ref4] Nishiyama Y.; Sugiyama J.; Chanzy H.; Langan P. Crystal structure and hydrogen bonding system in cellulose I(alpha) from synchrotron X-ray and neutron fiber diffraction. J. Am. Chem. Soc. 2003, 125, 14300–6. 10.1021/ja037055w. 14624578

[ref5] Nishiyama Y.; Langan P.; Chanzy H. Crystal structure and hydrogen-bonding system in cellulose Ibeta from synchrotron X-ray and neutron fiber diffraction. J. Am. Chem. Soc. 2002, 124, 9074–82. 10.1021/ja0257319. 12149011

[ref6] Tai H.-C.; Chang C.-H.; Cai W.; Lin J.-H.; Huang S.-J.; Lin Q.-Y.; et al. Wood cellulose microfibrils have a 24-chain core-shell nanostructure in seed plants. Nature Plants. 2023, 9, 1154–68. 10.1038/s41477-023-01430-z. 37349550

[ref7] Chen P.; Wohlert J.; Berglund L.; Furó I. Water as an Intrinsic Structural Element in Cellulose Fibril Aggregates. Journal of Physical Chemistry Letters. 2022, 13, 5424–30. 10.1021/acs.jpclett.2c00781. 35679323 PMC9234975

[ref8] Song B.; Zhao S.; Shen W.; Collings C.; Ding S.-Y. Direct Measurement of Plant Cellulose Microfibril and Bundles in Native Cell Walls. Frontiers in Plant Science. 2020, 11, 11 10.3389/fpls.2020.00479. 32391038 PMC7193091

[ref9] Ding S.-Y.; Zhao S.; Zeng Y. Size, shape, and arrangement of native cellulose fibrils in maize cell walls. Cellulose. 2014, 21, 863–71. 10.1007/s10570-013-0147-5.

[ref10] Langan P.; Petridis L.; O’Neill H. M.; Pingali S. V.; Foston M.; Nishiyama Y.; et al. Common processes drive the thermochemical pretreatment of lignocellulosic biomass. Green Chem. 2014, 16, 63–8. 10.1039/C3GC41962B.

[ref11] Phyo P.; Wang T.; Yang Y.; O’Neill H.; Hong M. Direct Determination of Hydroxymethyl Conformations of Plant Cell Wall Cellulose Using 1H Polarization Transfer Solid-State NMR. Biomacromolecules. 2018, 19, 1485–97. 10.1021/acs.biomac.8b00039. 29562125

[ref12] Zitting A.; Paajanen A.; Penttilä P. Impact of hemicelluloses and crystal size on X-ray scattering from atomistic models of cellulose microfibrils. Cellulose. 2023, 30, 8107–26. 10.1007/s10570-023-05357-8.

[ref13] Oehme D. P.; Yang H.; Kubicki J. D. An evaluation of the structures of cellulose generated by the CHARMM force field: comparisons to in planta cellulose. Cellulose. 2018, 25, 3755–77. 10.1007/s10570-018-1793-4.

[ref14] Emons A. M. C.. Role of particle rosettes and terminal globules in cellulose synthesis. In Biosynthesis and Biodegradation of Cellulose; Haigler C. H., Ed.; Marcel Dekker: New York, 1991; pp 71–98.

[ref15] Purushotham P.; Ho R.; Zimmer J. Architecture of a catalytically active homotrimeric plant cellulose synthase complex. Science 2020, 369, 1089–94. 10.1126/science.abb2978. 32646917

[ref16] Zhang X. N.; Xue Y.; Guan Z. Y.; Zhou C.; Nie Y. F.; Men S.; et al. Structural insights into homotrimeric assembly of cellulose synthase CesA7 from *Gossypium hirsutum*. Plant Biotechnol J. 2021, 19, 1579–87. 10.1111/pbi.13571. 33638282 PMC8384604

[ref17] Nixon B. T.; Mansouri K.; Singh A.; Du J.; Davis J. K.; Lee J. G.; et al. Comparative Structural and Computational Analysis Supports Eighteen Cellulose Synthases in the Plant Cellulose Synthesis Complex. Sci. Rep. 2016, 6, 28696 10.1038/srep28696. 27345599 PMC4921827

[ref18] Nicolas W. J.; Fassler F.; Dutka P.; Schur F. K. M.; Jensen G.; Meyerowitz E. Cryo-electron tomography of the onion cell wall shows bimodally oriented cellulose fibers and reticulated homogalacturonan networks. Curr. Biol. 2022, 32, 2375–89e6. 10.1016/j.cub.2022.04.024. 35508170 PMC9240970

[ref19] Xu P.; Donaldson L. A.; Gergely Z. R.; Staehelin L. A. Dual-axis electron tomography: a new approach for investigating the spatial organization of wood cellulose microfibrils. Wood Sci. Technol. 2007, 41, 101–16. 10.1007/s00226-006-0088-3.

[ref20] Lai-Kee-Him J.; Chanzy H.; Muller M.; Putaux J. L.; Imai T.; Bulone V. In vitro versus in vivo cellulose microfibrils from plant primary wall synthases: structural differences. Journal of biological chemistry. 2002, 277, 36931–9. 10.1074/jbc.M203530200. 12145282

[ref21] Deligey F.; Frank M. A.; Cho S. H.; Kirui A.; Mentink-Vigier F.; Swulius M. T.; et al. Structure of In Vitro-Synthesized Cellulose Fibrils Viewed by Cryo-Electron Tomography and (13)C Natural-Abundance Dynamic Nuclear Polarization Solid-State NMR. Biomacromolecules. 2022, 23, 2290–301. 10.1021/acs.biomac.1c01674. 35341242 PMC9198983

[ref22] Cho S. H.; Purushotham P.; Fang C.; Maranas C.; Diaz-Moreno S. M.; Bulone V.; et al. Synthesis and Self-Assembly of Cellulose Microfibrils from Reconstituted Cellulose Synthase. Plant Physiol. 2017, 175, 146–56. 10.1104/pp.17.00619. 28768815 PMC5580757

[ref23] Purushotham P.; Cho S. H.; Díaz-Moreno S. M.; Kumar M.; Nixon B. T.; Bulone V.; et al. A single heterologously expressed plant cellulose synthase isoform is sufficient for cellulose microfibril formation in vitro. Proceedings of the National Academy of Sciences. 2016, 113, 11360–5. 10.1073/pnas.1606210113. PMC5056052 27647898

[ref24] Davies L. M.; Harris P. J. Atomic force microscopy of microfibrils in primary cell walls. Planta. 2003, 217, 283–9. 10.1007/s00425-003-0979-6. 12783336

[ref25] Ding S. Y.; Himmel M. E. The maize primary cell wall microfibril: a new model derived from direct visualization. J. Agric. Food Chem. 2006, 54, 597–606. 10.1021/jf051851z. 16448156

[ref26] Marga F.; Grandbois M.; Cosgrove D. J.; Baskin T. I. Cell wall extension results in the coordinate separation of parallel microfibrils: evidence from scanning electron microscopy and atomic force microscopy. Plant Journal. 2005, 43, 181–90. 10.1111/j.1365-313X.2005.02447.x. 15998305

[ref27] Zhang T.; Zheng Y.; Cosgrove D. J. Spatial organization of cellulose microfibrils and matrix polysaccharides in primary plant cell walls as imaged by multichannel atomic force microscopy. Plant Journal. 2016, 85, 179–92. 10.1111/tpj.13102. 26676644

[ref28] Rongpipi S.; Ye D.; Gomez E. D.; Gomez E. W. Progress and Opportunities in the Characterization of Cellulose - An Important Regulator of Cell Wall Growth and Mechanics. Frontiers in Plant Science. 2019, 9, 1894 10.3389/fpls.2018.01894. 30881371 PMC6405478

[ref29] Rivnay J.; Mannsfeld S. C. B.; Miller C. E.; Salleo A.; Toney M. F. Quantitative Determination of Organic Semiconductor Microstructure from the Molecular to Device Scale. Chem. Rev. 2012, 112, 5488–519. 10.1021/cr3001109. 22877516

[ref30] Patterson A. L. The Scherrer Formula for X-Ray Particle Size Determination. Phys. Rev. 1939, 56, 978 10.1103/PhysRev.56.978.

[ref31] Rivnay J.; Noriega R.; Kline R. J.; Salleo A.; Toney M. F. Quantitative analysis of lattice disorder and crystallite size in organic semiconductor thin films. Phys. Rev. B 2011, 84, 045203 10.1103/PhysRevB.84.045203.

[ref32] Zhang W.; Bombile J. H.; Weisen A. R.; Xie R.; Colby R. H.; Janik M. J.; et al. Thermal Fluctuations Lead to Cumulative Disorder and Enhance Charge Transport in Conjugated Polymers. Macromol. Rapid Commun. 2019, 40, 1900134 10.1002/marc.201900134. 31116905

[ref33] Langford J. I.; Wilson A. Scherrer after sixty years: a survey and some new results in the determination of crystallite size. J. Appl. Crystallogr. 1978, 11, 102–13. 10.1107/S0021889878012844.

[ref34] Del Mundo J. T.; Rongpipi S.; Yang H.; Ye D.; Kiemle S. N.; Moffitt S. L.; et al. Grazing-incidence diffraction reveals cellulose and pectin organization in hydrated plant primary cell wall. Scientific Reports. 2023, 13, 5421 10.1038/s41598-023-32505-8. 37012389 PMC10070456

[ref35] Rongpipi S.; Ye D.; Gomez E. D.; Gomez E. W. Progress and Opportunities in the Characterization of Cellulose - An Important Regulator of Cell Wall Growth and Mechanics. Front Plant Sci. 2019, 9, 1894 10.3389/fpls.2018.01894. 30881371 PMC6405478

[ref36] Zhang F.; Skoda M. W. A.; Jacobs R. M. J.; Martin R. A.; Martin C. M.; Schreiber F. Protein Interactions Studied by SAXS: Effect of Ionic Strength and Protein Concentration for BSA in Aqueous Solutions. J. Phys. Chem. B 2007, 111, 251–9. 10.1021/jp0649955. 17201449

[ref37] Kikhney A. G.; Svergun D. I. A practical guide to small angle X-ray scattering (SAXS) of flexible and intrinsically disordered proteins. FEBS Lett. 2015, 589, 2570–7. 10.1016/j.febslet.2015.08.027. 26320411

[ref38] Penttilä P. A.; Paajanen A. Critical comment on the assumptions leading to 24-chain microfibrils in wood. Nat. Plants 2024, 10, 1064 10.1038/s41477-024-01689-w. 38769445

[ref39] Jakob H. F.; Tschegg S. E.; Fratzl P. Hydration Dependence of the Wood-Cell Wall Structure in Picea abies. A Small-Angle X-ray Scattering Study. Macromolecules. 1996, 29, 8435–40. 10.1021/ma9605661.

[ref40] Kennedy C. J.; Šturcová A.; Jarvis M. C.; Wess T. J. Hydration effects on spacing of primary-wall cellulose microfibrils: a small angle X-ray scattering study. Cellulose. 2007, 14, 401–8. 10.1007/s10570-007-9129-9.

[ref41] Fernandes A. N.; Thomas L. H.; Altaner C. M.; Callow P.; Forsyth V. T.; Apperley D. C.; et al. Nanostructure of cellulose microfibrils in spruce wood. Proc. Natl. Acad. Sci. U. S. A. 2011, 108, E1195–E203. 10.1073/pnas.1108942108. 22065760 PMC3223458

[ref42] Newman R. H. Estimation of the lateral dimensions of cellulose crystallites using 13C NMR signal strengths. Solid state nuclear magnetic resonance. 1999, 15, 21–9. 10.1016/S0926-2040(99)00043-0. 10903081

[ref43] Newman R. H.; Hill S. J.; Harris P. J. Wide-angle x-ray scattering and solid-state nuclear magnetic resonance data combined to test models for cellulose microfibrils in mung bean cell walls. Plant Physiol. 2013, 163, 1558–67. 10.1104/pp.113.228262. 24154621 PMC3846134

[ref44] Yang H.; Kubicki J. D. A density functional theory study on the shape of the primary cellulose microfibril in plants: effects of C6 exocyclic group conformation and H-bonding. Cellulose. 2020, 27, 2389–402. 10.1007/s10570-020-02970-9.

[ref45] Addison B.; Bu L.; Bharadwaj V.; Crowley M. F.; Harman-Ware A. E.; Crowley M. F.; et al. Atomistic, macromolecular model of the Populus secondary cell wall informed by solid-state NMR. Science Advances. 2024, 10, eadi7965 10.1126/sciadv.adi7965. 38170770 PMC10776008

[ref46] Doi Y.; Daicho K.; Isobe N.; Tanaka R.; Kimura S.; Fujisawa S.; et al. Monitoring crystallite fusion of nanocellulose during colloid condensation. Cellulose. 2023, 30, 8287–9. 10.1007/s10570-023-05354-x.

[ref47] Wickholm K.; Larsson P. T.; Iversen T. Assignment of non-crystalline forms in cellulose I by CP/MAS 13C NMR spectroscopy. Carbohydr. Res. 1998, 312, 123–9. 10.1016/S0008-6215(98)00236-5.

[ref48] Larsson P. T.; Hult E. L.; Wickholm K.; Pettersson E.; Iversen T. CP/MAS 13C-NMR spectroscopy applied to structure and interaction studies on cellulose I. Solid state nuclear magnetic resonance. 1999, 15, 31–40. 10.1016/S0926-2040(99)00044-2. 10903082

[ref49] Foster C. E.; Martin T. M.; Pauly M. Comprehensive compositional analysis of plant cell walls (lignocellulosic biomass) part II: carbohydrates. Journal of visualized experiments: JoVE 2010, na 10.3791/1837. PMC3145335 20228730

[ref50] Horii F; Hirai A; Kitamaru R. Cross-Polarization-Magic Angle Spinning Carbon-13 NMR Approach to the Structural Analysis of Cellulose. The Structures of Cellulose. ACS Symposium Series 340; American Chemical Society, 1987; pp 119–134.

[ref51] White P. B.; Wang T.; Park Y. B.; Cosgrove D. J.; Hong M. Water-polysaccharide interactions in the primary cell wall of Arabidopsis thaliana from polarization transfer solid-state NMR. J. Am. Chem. Soc. 2014, 136, 10399–409. 10.1021/ja504108h. 24984197

[ref52] Wang T.; Yang H.; Kubicki J. D.; Hong M. Cellulose Structural Polymorphism in Plant Primary Cell Walls Investigated by High-Field 2D Solid-State NMR Spectroscopy and Density Functional Theory Calculations. Biomacromolecules. 2016, 17, 2210–22. 10.1021/acs.biomac.6b00441. 27192562 PMC5270591

[ref53] Vietor R. J.; Newman R. H.; Ha M. A.; Apperley D. C.; Jarvis M. C. Conformational features of crystal-surface cellulose from higher plants. Plant J. 2002, 30, 721–31. 10.1046/j.1365-313X.2002.01327.x. 12061903

[ref54] Oehme D. P.; Downton M. T.; Doblin M. S.; Wagner J.; Gidley M. J.; Bacic A. Unique Aspects of the Structure and Dynamics of Elementary Iβ Cellulose Microfibrils Revealed by Computational Simulations. Plant Physiology. 2015, 168, 3–17. 10.1104/pp.114.254664. 25786828 PMC4424011

[ref55] Dupree R.; Simmons T. J.; Mortimer J. C.; Patel D.; Iuga D.; Brown S. P.; et al. Probing the Molecular Architecture of Arabidopsis thaliana Secondary Cell Walls Using Two- and Three-Dimensional (13)C Solid State Nuclear Magnetic Resonance Spectroscopy. Biochemistry. 2015, 54, 2335–45. 10.1021/bi501552k. 25739924

[ref56] Wickholm K.; Hult E. L.; Larsson P. T.; Iversen T.; Lennholm H. Quantification of cellulose forms in complex cellulose materials: a chemometric model. Cellulose. 2001, 8, 139–48. 10.1023/A:1016700325434.

[ref57] Kirui A.; Zhao W.; Deligey F.; Yang H.; Kang X.; Mentink-Vigier F.; et al. Carbohydrate-aromatic interface and molecular architecture of lignocellulose. Nature Communications. 2022, 13, 538 10.1038/s41467-022-28165-3. PMC8795156 35087039

[ref58] Simmons T. J.; Mortimer J. C.; Bernardinelli O. D.; Pöppler A.-C.; Brown S. P.; deAzevedo E. R.; et al. Folding of xylan onto cellulose fibrils in plant cell walls revealed by solid-state NMR. Nature Communications. 2016, 7, 13902 10.1038/ncomms13902. PMC5187587 28000667

[ref59] Wang T.; Hong M. Solid-state NMR investigations of cellulose structure and interactions with matrix polysaccharides in plant primary cell walls. J. Exp Bot. 2016, 67, 503–14. 10.1093/jxb/erv416. 26355148 PMC6280985

[ref60] Wilson T. H.; Kumar M.; Turner S. R. The molecular basis of plant cellulose synthase complex organisation and assembly. Biochem Soc. T. 2021, 49, 379–91. 10.1042/BST20200697. 33616627

[ref61] Kimura S.; Laosinchai W.; Itoh T.; Cui X.; Linder C. R.; Brown R. M. J. Immunogold labeling of rosette terminal cellulose-synthesizing complexes in the vascular plant Vigna angularis. Plant Cell. 1999, 11, 2075–86. 10.1105/tpc.11.11.2075. 10559435 PMC144118

[ref62] Herth W. Arrays of plasma-membrane ″rosettes″ involved in cellulose microfibril formation of Spirogyra. Planta. 1983, 159, 347–56. 10.1007/BF00393174. 24258233

[ref63] Giddings T. H. Jr.; Brower D. L.; Staehelin L. A. Visualization of particle complexes in the plasma membrane of Micrasterias denticulata associated with the formation of cellulose fibrils in primary and secondary cell walls. J. Cell Biol. 1980, 84, 327–339. 10.1083/jcb.84.2.327. 7189756 PMC2110545

[ref64] Herth W.; Weber G. Occurrence of the Putative Cellulose-Synthesizing Rosettes in the Plasma-Membrane of Glycine-Max Suspension-Culture Cells. Naturwissenschaften. 1984, 71, 153–4. 10.1007/BF01137780.

[ref65] Kimura S.; Laosinchai W.; Itoh T.; Cui X.; Linder C. R.; Brown R. M. Jr. Immunogold labeling of rosette terminal cellulose-synthesizing complexes in the vascular plant vigna angularis. Plant Cell. 1999, 11, 2075–2085. 10.1105/tpc.11.11.2075. 10559435 PMC144118

[ref66] Bowling A. J.; Brown R. M. Jr. The cytoplasmic domain of the cellulose-synthesizing complex in vascular plants. Protoplasma 2008, 233, 115–27. 10.1007/s00709-008-0302-2. 18709477

[ref67] Herth W. Plasma-membrane rosettes involved in localized wall thickening during xylem vessel formation of Lepidium sativum L. Planta. 1985, 164, 12–21. 10.1007/BF00391020. 24249494

[ref68] Haigler C. H.; Grimson M. J.; Gervais J.; Le Moigne N.; Höfte H.; Monasse B.; et al. Molecular modeling and imaging of initial stages of cellulose fibril assembly: evidence for a disordered intermediate stage. PloS one. 2014, 9, e93981 10.1371/journal.pone.0093981. 24722535 PMC3983097

[ref69] Zhang T.; Mahgsoudy-Louyeh S.; Tittmann B.; Cosgrove D. J. Visualization of the nanoscale pattern of recently-deposited cellulose microfibrils and matrix materials in never-dried primary walls of the onion epidermis. Cellulose. 2014, 21, 853–62. 10.1007/s10570-013-9996-1.

[ref70] Makarem M.; Lee C. M.; Kafle K.; Huang S. X.; Chae I.; Yang H.; et al. Probing cellulose structures with vibrational spectroscopy. Cellulose. 2019, 26, 35–79. 10.1007/s10570-018-2199-z.

[ref71] Kubicki J. D.; Mohamed M. N. A.; Watts H. D. Quantum mechanical modeling of the structures, energetics and spectral properties of I alpha and I beta cellulose. Cellulose. 2013, 20, 9–23. 10.1007/s10570-012-9838-6.

[ref72] Kubicki J. D.; Watts H. D.; Zhao Z.; Zhong L. H. Quantum mechanical calculations on cellulose-water interactions: structures, energetics, vibrational frequencies and NMR chemical shifts for surfaces of I alpha and I beta cellulose. Cellulose. 2014, 21, 909–26. 10.1007/s10570-013-0029-x.

[ref73] Kubicki J. D.; Yang H.; Sawada D.; O’Neill H.; Oehme D.; Cosgrove D. The shape of native plant cellulose microfibrils. Scientific Reports. 2018, 8, 13983 10.1038/s41598-018-32211-w. 30228280 PMC6143632

[ref74] Hill J. L. Jr.; Hammudi M. B.; Tien M. The Arabidopsis cellulose synthase complex: a proposed hexamer of CESA trimers in an equimolar stoichiometry. Plant Cell. 2015, 26, 4834–4842. 10.1105/tpc.114.131193. PMC4311198 25490917

[ref75] Adobes-Vidal M.; Frey M.; Keplinger T. Atomic force microscopy imaging of delignified secondary cell walls in liquid conditions facilitates interpretation of wood ultrastructure. J. Struct Biol. 2020, 211, 107532 10.1016/j.jsb.2020.107532. 32442716

[ref76] Fernando D.; Kowalczyk M.; Guindos P.; Auer M.; Daniel G. Electron tomography unravels new insights into fiber cell wall nanostructure; exploring 3D macromolecular biopolymeric nano-architecture of spruce fiber secondary walls. Sci. Rep. 2023, 13, 2350 10.1038/s41598-023-29113-x. 36759530 PMC9911387

[ref77] Lyczakowski J. J.; Bourdon M.; Terrett O. M.; Helariutta Y.; Wightman R.; Dupree P. Structural Imaging of Native Cryo-Preserved Secondary Cell Walls Reveals the Presence of Macrofibrils and Their Formation Requires Normal Cellulose, Lignin and Xylan Biosynthesis. Front Plant Sci. 2019, 10, 1398 10.3389/fpls.2019.01398. 31708959 PMC6819431

[ref78] Zhang T.; Zheng Y. Z.; Cosgrove D. J. Spatial organization of cellulose microfibrils and matrix polysaccharides in primary plant cell walls as imaged by multichannel atomic force microscopy. Plant Journal. 2016, 85, 179–92. 10.1111/tpj.13102. 26676644

[ref79] Ding S. Y.; Himmel M. E. The maize primary cell wall microfibril: A new model derived from direct visualization. J. Agric. Food Chem. 2006, 54, 597–606. 10.1021/jf051851z. 16448156

[ref80] Ding S. Y.; Zhao S.; Zeng Y. N. Size, shape, and arrangement of native cellulose fibrils in maize cell walls. Cellulose. 2014, 21, 863–71. 10.1007/s10570-013-0147-5.

[ref81] Brown R. M.; Willison J. H.; Richardson C. L. Cellulose biosynthesis in Acetobacter xylinum: visualization of the site of synthesis and direct measurement of the in vivo process. Proc. Natl. Acad. Sci. U. S. A. 1976, 73, 4565–9. 10.1073/pnas.73.12.4565. 1070005 PMC431544

[ref82] Babi M.; Williams A.; Reid M.; Grandfield K.; Bassim N. D.; Moran-Mirabal J. M. Unraveling the Supramolecular Structure and Nanoscale Dislocations of Bacterial Cellulose Ribbons Using Correlative Super-Resolution Light and Electron Microscopy. Biomacromolecules. 2023, 24, 258–68. 10.1021/acs.biomac.2c01108. 36577132

[ref83] Abidi W.; Decossas M.; Torres-Sanchez L.; Puygrenier L.; Letoffe S.; Ghigo J. M.; et al. Bacterial crystalline cellulose secretion via a supramolecular BcsHD scaffold. Sci. Adv. 2022, 8, eadd1170 10.1126/sciadv.add1170. 36525496 PMC9757748

[ref84] Sugiyama J.; Harada H.; Fujiyoshi Y.; Uyeda N. Lattice images from ultrathin sections of cellulose microfibrils in the cell wall of *Valonia macrophysa* Kutz. Planta. 1985, 166, 161–8. 10.1007/BF00397343. 24241427

[ref85] Tsekos I. The sites of cellulose synthesis in algae: Diversity and evolution of cellulose-synthesizing enzyme complexes. J. Phycol. 1999, 35, 635–55. 10.1046/j.1529-8817.1999.3540635.x.

[ref86] Haigler C. H.; Roberts A. W. Structure/function relationships in the rosette cellulose synthesis complex illuminated by an evolutionary perspective. Cellulose. 2019, 26, 227–47. 10.1007/s10570-018-2157-9.

[ref87] Cosgrove D. J. Structure and growth of plant cell walls. Nat. Rev. Mol. Cell Bio 2024, 25, 340 10.1038/s41580-023-00691-y. 38102449

[ref88] Haigler C. H.; White A. R.; Brown R. M. Jr.; Cooper K. M. Alteration of in vivo cellulose ribbon assembly by carboxymethylcellulose and other cellulose derivatives. J. Cell Biol. 1982, 94, 64–69. 10.1083/jcb.94.1.64. 6889605 PMC2112193

[ref89] Chibrikov V.; Pieczywek P. M.; Zdunek A. Tailor-Made Biosystems-Bacterial Cellulose-Based Films with Plant Cell Wall Polysaccharides. Polym. Rev. 2023, 63, 40–66. 10.1080/15583724.2022.2067869.

[ref90] Jarvis M. C. Hydrogen bonding and other non-covalent interactions at the surfaces of cellulose microfibrils. Cellulose. 2023, 30, 667–87. 10.1007/s10570-022-04954-3.

[ref91] Lee C. M.; Kafle K.; Belias D. W.; Park Y. B.; Glick R. E.; Haigler C. H.; et al. Comprehensive analysis of cellulose content, crystallinity, and lateral packing in and cotton fibers using sum frequency generation, infrared and Raman spectroscopy, and X-ray diffraction. Cellulose. 2015, 22, 971–89. 10.1007/s10570-014-0535-5.

[ref92] Avci U.; Pattathil S.; Singh B.; Brown V. L.; Hahn M. G.; Haigler C. H. Cotton fiber cell walls of *Gossypium hirsutum* and *Gossypium barbadense* have differences related to loosely-bound xyloglucan. PloS one. 2013, 8, e56315 10.1371/journal.pone.0056315. 23457548 PMC3572956

[ref93] Meinert M. C.; Delmer D. P. Changes in biochemical composition of the cell wall of the cotton fiber during development. Plant Physiol. 1977, 59, 1088–97. 10.1104/pp.59.6.1088. 16660000 PMC542513

[ref94] Martinez-Sanz M.; Pettolino F.; Flanagan B.; Gidley M. J.; Gilbert E. P. Structure of cellulose microfibrils in mature cotton fibres. Carbohydr. Polym. 2017, 175, 450–63. 10.1016/j.carbpol.2017.07.090. 28917888

[ref95] Wen X.; Zhai Y.; Zhang L.; Chen Y.; Zhu Z.; Chen G.; et al. Molecular studies of cellulose synthase supercomplex from cotton fiber reveal its unique biochemical properties. Sci. China Life Sci. 2022, 65, 1776–93. 10.1007/s11427-022-2083-9. 35394636

[ref96] Ono Y.; Hou G. Y.; Chitbanyong K.; Takeuchi M.; Isogai A. Molar masses and molar mass distributions of commercial regenerated cellulose materials and softwood dissolving pulp determined by SEC/MALLS. Cellulose. 2023, 30, 8221–33. 10.1007/s10570-023-05414-2.

[ref97] McFarlane H. E. Open questions in plant cell wall synthesis. Journal of Experimental Botany. 2023, 74, 3425–48. 10.1093/jxb/erad110. 36961357

[ref98] Delmer D.; Dixon R. A.; Keegstra K.; Mohnen D. The plant cell wall-dynamic, strong, and adaptable-is a natural shapeshifter. Plant Cell. 2024, 36, 1257–311. 10.1093/plcell/koad325. 38301734 PMC11062476

